# Materials Testing for the Development of Biocompatible Devices through Vat-Polymerization 3D Printing

**DOI:** 10.3390/nano10091788

**Published:** 2020-09-09

**Authors:** Gustavo González, Désirée Baruffaldi, Cinzia Martinengo, Angelo Angelini, Annalisa Chiappone, Ignazio Roppolo, Candido Fabrizio Pirri, Francesca Frascella

**Affiliations:** 1 Dipartimento di Scienza Applicata e Tecnologia, Politecnico di Torino, C.so Duca degli Abruzzi 24, 10129 Turin, Italy; gustavo.gonzalez@polito.it (G.G.); desiree.baruffaldi@polito.it (D.B.); cinzia.martinengo@polito.it (C.M.); annalisa.chiappone@polito.it (A.C.); ignazio.roppolo@polito.it (I.R); fabrizio.pirri@polito.it (C.F.P.); 2 Center for Sustainable Futures @Polito, Istituto Italiano di Tecnologia, Via Livorno 60, 10144 Turin, Italy; 3 PolitoBIOMed Lab, Politecnico di Torino, C.so Duca degli Abruzzi 24, 10129 Turin, Italy; 4 Advanced Materials Metrology and Life Sciences Division, Istituto Nazionale di Ricerca Metrologica, Strada delle Cacce 91, 10135 Torino, Italy; angelo.angelini@polito.it

**Keywords:** 3D printing, photopolymerization, microfluidics, biomaterials, cytotoxicity, cell culture

## Abstract

Light-based 3D printing techniques could be a valuable instrument in the development of customized and affordable biomedical devices, basically for high precision and high flexibility in terms of materials of these technologies. However, more studies related to the biocompatibility of the printed objects are required to expand the use of these techniques in the health sector. In this work, 3D printed polymeric parts are produced in lab conditions using a commercial Digital Light Processing (DLP) 3D printer and then successfully tested to fabricate components suitable for biological studies. For this purpose, different 3D printable formulations based on commercially available resins are compared. The biocompatibility of the 3D printed objects toward A549 cell line is investigated by adjusting the composition of the resins and optimizing post-printing protocols; those include washing in common solvents and UV post-curing treatments for removing unreacted and cytotoxic products. It is noteworthy that not only the selection of suitable materials but also the development of an adequate post-printing protocol is necessary for the development of biocompatible devices.

## 1. Introduction

Three-dimensional (3D) printing technology is a fundamental pillar of the so-called industry 4.0, leading changes in product design and manufacturing [1]. Compared to conventional methods, such as machining and mold-casting, the interest in this technology lies in the ease to create complex structures with customizable and multifunctional properties [2,3,4]. 3D printing is based on a layer-by-layer process: the object is produced through a sequential deposition of materials following a computer-aided design (CAD). This results in a more efficient use of raw materials, producing reduced waste, and lowering energy losses [5,6,7].

Within the 3D printing field, one of the most developed categories is polymeric 3D printing. The techniques for polymer manufacturing offer affordable costs of equipment as well as a wide range of commercially available polymers [8,9]. These conditions make polymeric 3D printing technology an ideal candidate for producing cost-effective and on-demand parts for biomedical applications [10,11]. It was already reported that this technology allows the manufacturing of bespoke structures for the fabrication of high-throughput polymer platforms that can be used in a vast range of bioengineering applications, such as non-planar lab-on-a-chip (LOC) [12,13,14], bioreactors for real-time analysis [15], and analytical systems with antibacterial properties for studying cell responses [16]. In this context, the light-based polymeric 3D printing technologies, such as stereolithographic (SL), digital light processing (DLP), and two-photon polymerization (2PP) are well-positioned, since they offer the possibility to tailor the final properties of the pieces by operating with both the starting printable materials and the printing settings [17,18]. Their operative principle is based on the spatially-controlled solidification of liquid photocurable resins upon light irradiation; those techniques show higher printing resolution and faster printing times compared to other polymeric printing techniques, such as fused filament fabrication (FFF), selective laser melting (SLM), and inkjet printing [19,20]. Commonly, the photocurable resins for 3D printing are composed of three main components: (i) the monomer/oligomer and liquid/viscous resins bearing reactive groups necessary to form the polymeric network. The backbone of the monomers defines the final physical and mechanical properties of the part. Regarding the photoactive moieties, most of the used reactive groups are acrylate or methacrylate due to the fast reaction rate [21]. (ii) The photoinitiator, which is necessary to initiate the reaction through light absorption. (iii) A dye or colorant, which is added to control the penetration of the light during 3D printing (Z-axis) and to guarantee high resolution (XY plane). Considering the high availability of raw materials, light-based technologies may allow the manufacturing of customizable and complex-shaped 3D printed polymeric parts just by selecting the appropriate components [22,23]. However, most of the actual available materials do not meet the primary requirements in bioengineering field (e.g., cell culturing or tissue engineering), such as transparency, water, and gas permeability, flexibility, and, most of all, biocompatibility. In fact, despite the versatility and the above-mentioned advantages of 3D printing by photopolymerization, its use in the biological field is limited by the low biocompatibility of the polymeric parts, mainly due to the presence of unreacted products after the polymerization (monomer, photoinitiator, and additives) [24,25,26]. Hence, there is a growing interest in recent times for the development of biocompatible (meth)acrylate resins for light-based 3D printing applications [27,28]. Regarding biocompatible acrylate-based 3D printed devices, it has recently been reported the preparation of formulations using acrylate monomers for the 3D printing of objects for Human Umbilical Vein Endothelial Cells (HUVECs) culturing with moderate viability [29]. Urrios et al. used a low-molecular PEG-based acrylate monomer for the production of 3D printed transparent bio-microfluidics devices and Petri-dish platforms used for mammalian cells and hippocampal neurons culture [30].

However, to further expand the applicability and resourcefulness of light-based 3D printing techniques, more extensive investigations for comprehending the cytocompatibility of the photocurable resins are required. Therefore, in this manuscript, we aim at producing biocompatible and transparent 3D printed polymer parts using a commercial DLP-3D printer for testing formulations based on some of the most diffused resins for 3D printing. For this purpose, 96- and 24-wells-like polymeric parts were fabricated and tested. As acrylate monomers, polyethylene glycol diacrylate (PEGDA) [31,32], 1,6-hexanediol diacrylate (HDDA) [33,34], and bisphenol A ethoxylate diacrylate (BEDA) [35,36] were employed; as a photoinitiator, a phosphine oxide-based compound (BAPO) was selected. This photoinitiator is widely employed in SL or DLP 3D printing [37,38,39] but it is not considered biocompatible, unlike other photoinitiators such as Lithium phenyl–2,4,6–trimethylbenzoylphosphinate (LAP). However BAPO is 20 times cheaper than LAP and it was demonstrated that it may show low toxicity effects on cells if added in the adequate concentrations [30,40,41]. Some of the materials selected for this work have been previously reported as components of commercial light-3D printable resins such as E-Dent 100 (Envisiontec) or MED620 (Stratasys), which have been approved by the Food and Drugs Association (FDA) from the United States of America and/or CE-certified from European union (ISO 13485) [42,43,44]. However, this study aims to report not only the role of the materials, but also the key-effect of different post-printing protocols. Thus, by selecting the right materials and performing an accurate post-printing protocol, these home-made resins can be used for the manufacture of devices in biological fields [27,45,46].

## 2. Materials and Methods 

### 2.1. Materials 

Polyethylene glycol diacrylate (PEGDA, Mn = 250 g/mol), 1,6-hexanediol diacrylate (HDDA, Mw = 226.3 g/mol) and bisphenol A ethoxylate diacrylate (BEDA, Mn ≈ 512 g/mol, EO/phenol 2) were used as diacrylate reactive monomers. Phenyl bis(2,4,6–trimethylbenzoyl)-phosphine oxide (BAPO) was used as a photoinitiator. All chemical solvents and reagents were obtained from Merck Company (Darmstadt, Germany) and used as received. The formulations were prepared by adding a certain quantity of BAPO photoinitiator into each acrylate monomer. Then, the mixtures were sonicated for 30 min. In total, six formulations were prepared, their composition and nomenclature are shown in Table 1.

### 2.2. Digital Light Processing 3D Printing

A PICO 2 DLP-3D printer (Asiga, Australia) was used for processing of the acrylate-based formulations. This 3D printer has a LED light source (405 nm), the nominal XY pixel resolution is 50 μm, the minimum *Z*-axis control is 1 μm. The structures were 3D printed by setting a printing slicing of 50 µm, a light intensity of 20 mW/cm^2^. The irradiation times used for each formulation are shown in Tabel 1. Afterward, the printed objects were cleaned up, following the washing and UV post-curing protocols detailed below. 

### 2.3. Washing and UV Post-Curing Protocols

To optimize the cytocompatibility of the objects, the 3D printed wells obtained from each formulation, were deeply washed with ethanol or acetone for the complete removal of unreacted products. We tested four different washing protocols, identifiable into two main groups, detailed as follows: (1) sonication: the samples were sonicated for 5 min in a solvent (ethanol or acetone), then UV post-curing was performed irradiating the inner part of the well for 5 min, followed by another 5 min of irradiation of the outer part. After the post-curing, the samples were kept in the indicated solvent overnight (o/n). (2) incubation: the samples were incubated for 2 h in the indicated solvent (ethanol or acetone); then UV post-curing was performed as mentioned above.

### 2.4. Sterilization Protocol

Three different sterilization methods were performed to investigate the impact of each of the 3D printed samples and thus to select the most suitable method for our purpose. The 3D printed samples were sterilized (1) with UV light for 30 min inside a biosafety cabinet; (2) with immersion in 70% ethanol for 10 min, (3) autoclaved for 20 min at 121 °C, then the parts were air-dried at room temperature. Live/dead fluorescence assays (Sigma Aldrich, Saint Louis, MO, USA) were performed to evaluate the number of viable cells in culture.

### 2.5. Characterization Methods

Viscosity measurements of the formulations were performed using an Anton Paar rheometer (Physica MCR 302, Graz, Australia) at a constant temperature of 25 °C and in parallel-plate mode with a gap of 50 µm between two aluminum plates (both with a diameter of 20 mm). The shear rate range was set up from 1 to 100 s^−1^.

Real-time photo-rheological tests were performed to measure the changes in the viscoelastic properties during polymerization. The experiments were carried out using a 20 mm quartz lower parallel plate and a 20 mm aluminum upper parallel plate; a Hamamatsu LC8 lamp with a visible bulb with a cutoff filter below 400 nm and equipped with an 8 mm light guide was used to study the photopolymerization process. During the tests, the light irradiation started after 60 seconds from the beginning of the test to stabilize the system (light intensity set at 20 mW/cm^2^); the gap between the two parallel plates was set at 50 µm. The measurements were performed at a constant temperature of 25 °C, at constant strain amplitude of 1%, and a constant shear frequency of 1 rad/s. 

UV−Vis spectroscopy measurements were conducted using a Synergy™ HTX Multi-Mode Microplate Reader instrument (BioTek, Winooski, VM, USA) set in spectrum mode in the range between 300 to 700 nm and at a scan step of 10 nm. The experiments on solid films were performed on 3D printed discs (thickness 50 μm, diameter 20 mm) and using a 6-well plate. The UV-vis instrument was also used for analyzing the chloroform solutions used for evaluating the insoluble fraction.

The insoluble fraction (gel content) of the printed samples was determined following the standard test method ASTM D2765−84. The samples were held in a metal net, accurately weighed, and subsequently submitted to extraction with chloroform (CHCl_3_) for 24 h at room temperature to dissolve the unreacted products. At last, the samples were dried overnight at 80 °C, and the insoluble fraction percentage was determined as weight difference before and after solvent extraction. 

A Nicolet iS50 FT-IR spectrometer (Thermo Scientific, Milano, IT) was used to investigate the acrylate conversion on both liquid formulations and the 3D printed parts. The spectra were recorded using an attenuated total reflectance (ATR) accessory (Smart iTX). For the FTIR experiments, the spectra were collected with a resolution of 4 cm^−1^, averaging 32 scans for each spectrum, wavenumbers range 650–4000 cm^−1^. The conversion of the acrylate double bond (C=C) was monitored by the scissoring acrylate vibration at 1409 cm^−1^. The area of the acrylate peaks was normalized by the carbonyl (C=O) constant signal centered at 1725 cm^−1^. 

A microscope (Eclipse Ti2 Nikon, Tokyo, Japan) equipped with a Crest X-Light spinning disk confocal microscope and a Lumencor SPECTRA X light engine was used for the collection of bright-field and fluorescence images. All images were displayed using the same scaling and were collected using a Plan Apo 20 × 0.75 NA (Nikon, Tokyo, Japan).

Environmental Scanning Electron Microscopy (ESEM) images were collected through FEI Quanta 3D FEG Dual Beam Microscope (ThermoFisher, Waltham, MA, USA). This instrument enables us to operate in ambient conditions, avoiding the metallization of the sample. In detail, samples were observed at 5 kV and a chamber pressure of 60 millibars of H_2_O vapor. The cells were seeded into the 3D printed wells (inner diameter = 16.5 mm, cell number = 100,000), and after 48 h, the samples (with cells) were prepared for SEM microscopy. The samples were washed twice with PBS, then they were incubated with 2% glutaraldehyde in PBS for 1 h at room temperature. After this incubation, the samples were dehydrated using increasing amounts of ethanol: water (50%, 70%, 85%, and 100%) for 15 min each. At last, the samples were air-dried.

### 2.6. Cell Culture

For cell culture studies, lung cancer epithelial cells A549, kindly provided by Valentina Monica, from the Department of Oncology, University of Torino, AOU San Luigi Gonzaga, were maintained in RPMI 1640 medium, supplemented with 10% fetal bovine serum, 1% penicillin/streptomycin (all from Sigma Aldrich) and 2 mM glutamine (Biowest). When needed, cell morphologies were assessed with a phase-contrast microscope (DMi1, Leica Microsystems GmbH).

### 2.7. Cell Viability and Cell Proliferation Assays 

Before seeding cells, all samples were incubated o/n in deionized water at room temperature and, then, sterilized 30 min under UV light of biological hood. 

For live and dead staining assay 1 × 10^5^ A549 were seeded in each 3D-printed well, and the viability was assessed after 72 h. In particular, cells were washed three times with PBS, then they were stained with 1.5 μM Propidium Iodide (PI) and 1 μM Calcein-AM for 15 min at room temperature. Fluorescence was detected with a spinning disk confocal microscopy system. Cells were also seeded and analyzed in a conventional PS well as a control.

For MTT assay, 1.5 × 10^4^ A549 were seeded on each sample and incubated in complete medium at 37 °C. After 24 and 48 h of incubation, the medium was removed and 125 μL of fresh medium and 125 μL of 1 mg/mL MTT (dissolved in PBS) were added in each well and incubated for 2 h at 37 °C. After incubation, 250 μL of MTT solvent (10% SDS, 0.01 M HCl in H2O) was added to solubilize the formazan crystals and the plate was incubated for 2 h at 37 °C. Synergy™ HTX Multi-Mode Microplate Reader (BioTek, Winooski, VM, USA) was used to read the optical density (OD) at a wavelength of 570 and 650 nm. Blank media without the polymers were used as a control. The colorimetric signal (absorbance) is proportional to the number of proliferating cells. Error bars show mean SD.

## 3. Results and Discussion 

### 3.1. 3D printing and Material Characterizations 

Some of the acrylate monomers and the photoinitiator used in this work have been employed in light-based 3D printing with inherent biocompatible features [42,43,44], however, to study the biocompatibility of the 3D printed parts, different post-printing protocols were evaluated to remove the potentially toxic unreacted products.

Initially, a series of preliminary tests were performed. Those include viscosity, reactivity, and degree of conversion. The first feature that could compromise the printability of resins is the viscosity. As demonstrated in previous investigations, high viscosity resins could hinder the fabrication process of the objects in the printing step [47,48]. Accordingly, photosensitive resins with relatively low viscosity are favorable for fast and accurate fabrication of solid three-dimensional pieces. Although PEGDA, HDDA, and BEDA monomers have been widely used for light-based 3D printing, we briefly evaluated the viscosity of all formulations, data reported in Table 1. Viscosity for PEGDA and HDDA monomers (at both BAPO concentrations) are similar and lower if compared to BEDA monomer. However, as expected, such values are all suitable for vat polymerization techniques (SL or DLP) [33,49,50]. 

**Figure 1:**
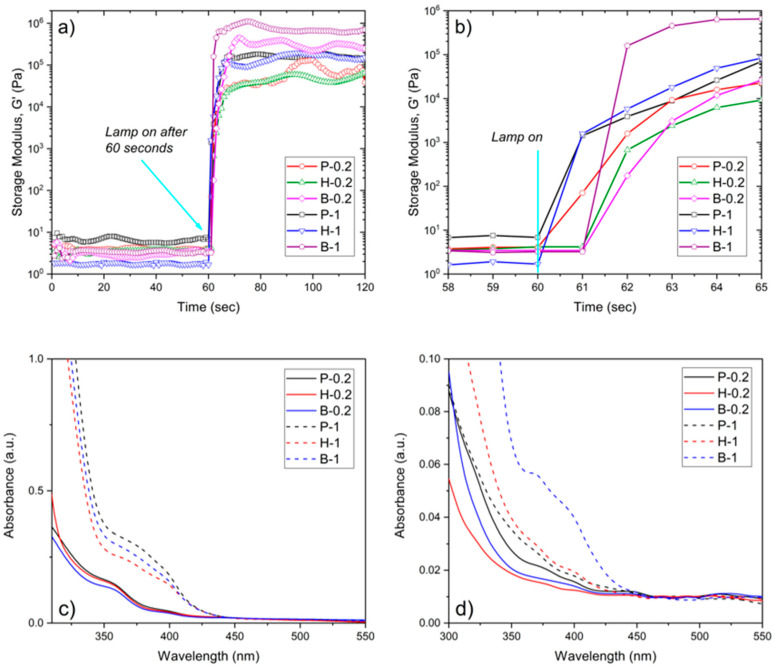
(**a**) Storage modulus (G’) as a function of irradiation time (Intensity 20 mW/cm^2^) for PEGDA, BEDA, and HDDA formulations containing 0.2 and 1 wt.% of BAPO. (**b**) Zoom of the G’ vs. irradiation time after the visible lamp is on. (**c**) UV-vis spectra of 100 µm thick 3D printed discs made of PEGDA, BEDA, and HDDA formulations with 0.2 wt.% (continue lines) and 1 wt.% (dashed line) of BAPO. (**d**) UV-Vis spectra of the extracting solvent used for the calculation of the insoluble gel fraction of the 3D printed parts.

The reactivity of the photosensitive resins was evaluated by means of real-time photorheology, measuring the changes in viscoelastic properties; specifically, the storage modulus (G’), against irradiation times. In Figure 1a, the G’ curves relative to the PEGDA, HDDA, and BEDA monomers, each loaded with both 0.2 wt.% and 1 wt.% of the BAPO photoinitiator, are reported. Once the visible lamp is switched on, all formulations present high reactivity with a sudden increase of G’ in the first 2 seconds. A slight delay of the reaction onset was visible for those formulations with lower photoinitiator content, as expected. This delay is better appreciated in Figure 1b. Therefore, formulations with the lower amount of BAPO (P-0.2, H-0.2, and B-0.2) may require longer exposure times during the printing step to cure a layer of the same thickness than those formulations with a higher BAPO amount (P-1, H-1, and B-1).

**Figure 2:**
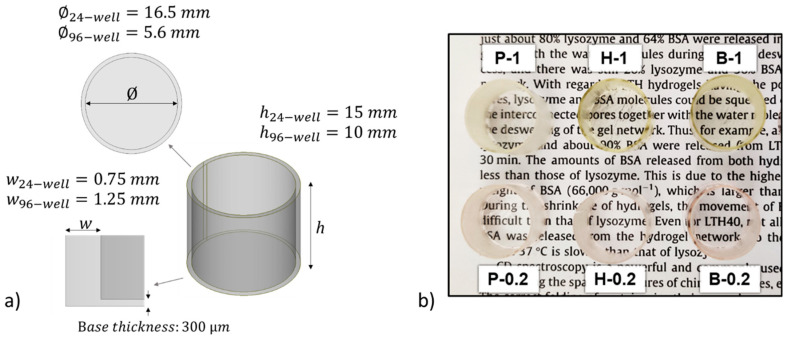
(**a**) Isometric view of the CAD designs showing the dimensions for 24- and 96-like-well fabrication. (**b**) Photograph of 3D printed 24-well-like objects from each prepared home-made resin.

The information collected from these preliminary tests was used to proceed to the 3D printing step. 96- and 24-like-wells were successfully printed using the printing parameter reported in Table 1. The dimensions of the objects are reported in Figure 2a. Figure 2b shows the photograph of 24-like 3D printed wells. Noteworthy is the more yellow hue of samples with 1 wt.% of BAPO compared to those with 0.2 wt.% of BAPO, which is caused by the higher amount of photoinitiator. Therefore, the transparency of the 3D printed wells at both BAPO concentrations was further evaluated using a microplate reader set up in the UV-vis scanning mode (results shown in Figure 1c). As expected, the samples with the higher concentration of photoinitiator (P-1, H-1, and B-1) showed a broad absorption band between 350 and 430 nm, which is related to BAPO. Even though the samples with the higher BAPO concentration displayed good optical characteristics (with the possibility of reading through the bottom of the well in Figure 2b), the slight yellow coloration exhibited could hinder the correct reading of the cells in the downstream studies. Furthermore, an excess concentration of the BAPO photoinitiator could also have toxic effects on the cells under study as demonstrated elsewhere [37,38].

The 3D printed wells were further characterized by using the ATR-FTIR technique and monitoring the decrease of the acrylate double bonds at 1409 cm^−1^ band related to the CH_2_ scissoring vibration. The acrylate conversion for each type of sample was calculated and reported in Table 1. Samples containing a higher BAPO amount (P-1, H-1, and B-1) reached a higher degree of conversion after the 3D printing step. An expected behavior considering the greater number of free radicals generated within the system upon visible irradiation during 3D printing, increasing the rate of initiation. Comparing monomers, the BEDA-based resins showed the lowest acrylate conversions, due to topological factors, such as molecular weight, higher viscosity, and the higher glass transition temperature [13]. The insoluble fraction amount of 3D printed samples was also evaluated by chloroform extraction for 24 h, results are reported in Table 1. Despite the differences in acrylate conversion, the insoluble fraction of the printed samples resulted to be similar and quite high for all of them, with only a slightly lower value for samples with 0.2 wt.% of BAPO. It is worthy to highlight that the extracted weight can belong both to unreacted monomers and to the photoinitiator. Both elements may result cytotoxic, therefore, it would be preferable to get rid of this component. This will be investigated more in detail considering the post-processing step.

The solvent used for the insoluble fraction tests (chloroform) was subsequently analyzed through UV-visible spectroscopy to evaluate the presence of any unreacted products released from the 3D printed samples, results plotted in Figure 1d. As observed, an absorption band appears around 350 to 430 nm (associated with BAPO) for samples with the highest photoinitiator concentration, indicating that some unreacted photoinitiator was present. This results in a higher content of unreacted products after the printing step, particularly for B-1 samples.

### 3.2. Cytotoxic Effect on A549 

**Figure 3:**
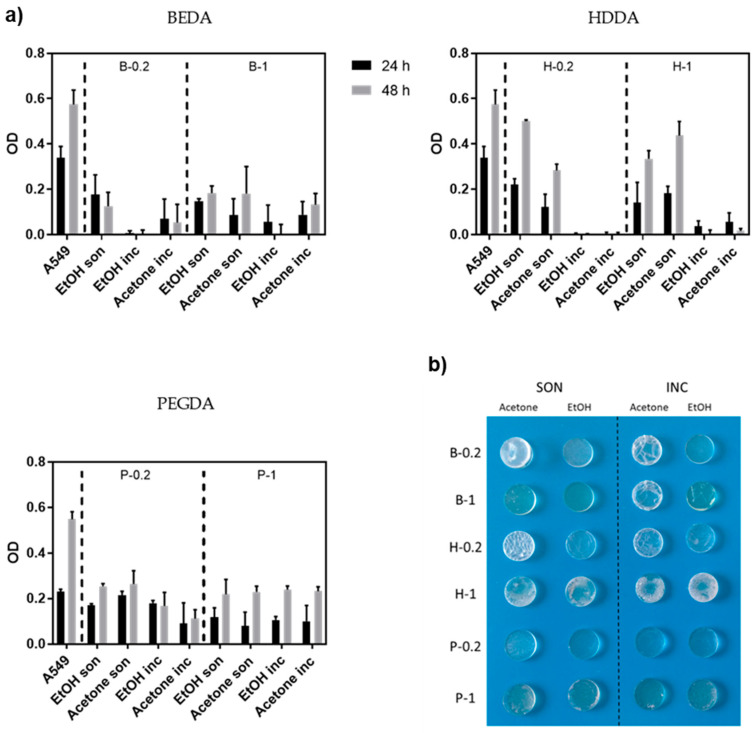
(**a**) Measurement of growth (MTT assay) of A549 cells seeded on three different resins (BEDA, HDDA, and PEGDA) each with two different amount of photoinitiator (0.2 wt.% and 1 wt.%, respectively) and four different washing protocols (i.e., ethanol sonicated, acetone sonicated, ethanol incubated, and acetone incubated), as previously described. The viability is measured after 24 and 48 h. Error bars show mean SD. (**b**) Photograph showing the optical and mechanical properties of 3D printed disks made of the same three resins and four washing protocols evaluated by MTT assay.

The MTT assay was performed to evaluate cell proliferation on 3D printed samples based on the material used and the post-printing process for removing the non-polymerized products (monomers and photoinitiators). Cells seeded in PS wells were considered as the positive control. The 3D printed samples were washed and sterilized, following the specific protocols described in the Materials and Methods section, then 1.5 × 10^4^ A549 were seeded on each sample and cultured for 48 h (Figure 3).

All the samples showed no big differences in A549 proliferation after 48 h, independently by the BAPO amount. This is most likely due to the effectiveness of the washing steps, which allow for the removal of the unreacted products. Nevertheless, we decided to continue the experiments with the samples with 0.2 wt.% of BAPO in order to use less reagent and to minimize possible BAPO toxicity, which is reported by previous works [40]. Focusing on samples with 0.2 wt.% of BAPO, the cell viability differs greatly depending on the starting acrylate resin used. The cell viability after 24 h was quite low for B-0.2 regardless of the washing protocols used, better results were obtained for H-0.2 and P-0.2 based 3D-printed cell culture samples. In particular, samples subjected to the washing protocols previously defined as sonication (i.e., 5 min sonication in solvent + 10 min UV post-curing + o/n in a solvent) showed a more pronounced proliferation between 24 and 48 h compared to incubation. This trend indicates that the cells were viable, and the 3D-printed substrates resulted non-cytotoxic. However, even if the MTT assay showed that the sonicated H-0.2 samples were the best materials, their low resistance to the solvents during washing made us lean towards selecting the P-0.2 for the following analysis. A collection of the real images of 3D printed disks of the various formulations that have undergone the different washing protocols, are clearly shown in Figure 3b. Comparing the P-0.2 samples sonicated in ethanol or acetone, we preferred the ethanol ones, because of the great optical transparency obtained after the washing step, which is a crucial characteristic for optical monitoring.

**Figure 4:**
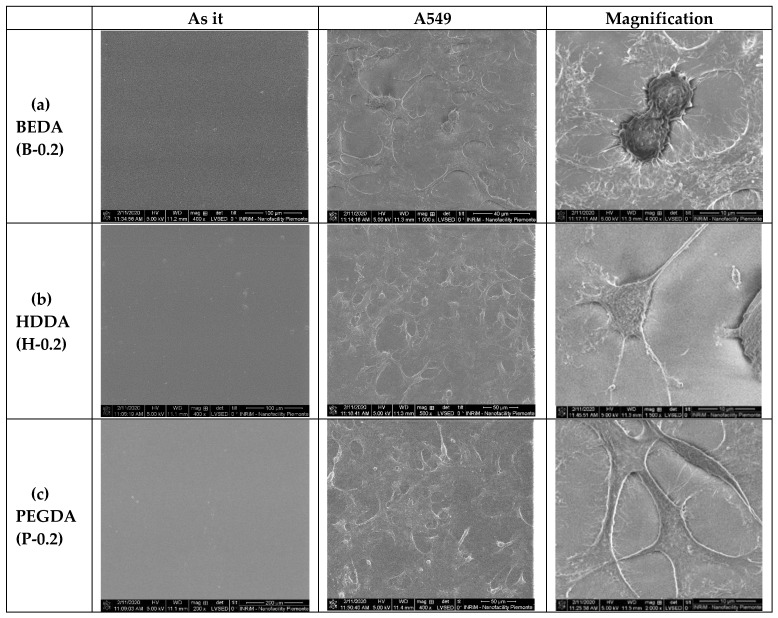
Environmental Scanning Electron Microscope (ESEM) images of the A549 cells cultured on the three different acrylate samples (**a**) BEDA, B-0.2, (**b**) HDDA, H-0.2, (**c**) PEGDA, P-0.2. For each formulation, three different SEM images are shown, in particular, the polymer as it is without cells and the same polymer with A549 at different degree of magnification (scale bar 100, 50, and 10 microns, respectively).

In addition to the biocompatibility method, the A549 cell morphology was studied on the 3D printed wells (PEGDA, HDDA, and BEDA monomers with 0.2 wt.% of BAPO) by Environmental Scanning Electron Microscopy (ESEM). The ESEM images (Figure 4) demonstrated the different degree of biocompatibility of the three acrylate-based samples, as already shown by the cytotoxic assay. In particular, the B-0.2 wells showed cells with severe changes in morphology concerning to the classical epithelial-like shape of A549 cells. In the B-0.2 samples, most of the cells appeared round and spongy. The cells morphology on the other two types of 3D printed wells (H-0.2 and P-0.2) are quite similar, both presented cells with epithelial appearance, well-attached to the surface of the 3D printed structure and with no evident signs of apoptosis; even if for P-0.2 this aspect seemed to be more evident.

**Figure 5:**
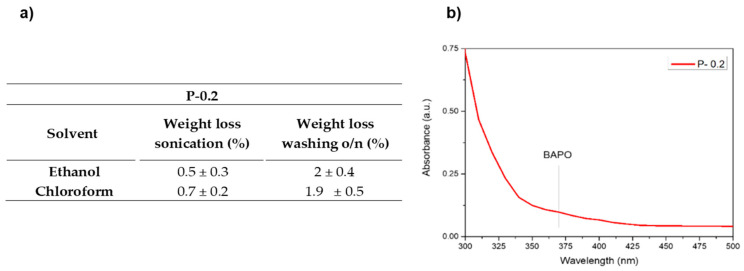
(**a**) Weight losses after the different washing steps in ethanol and chloroform for sample P-0.2. (**b**) UV-Vis spectrum of the washing ethanol after the o/n step.

Aiming to further check the efficiency of the selected washing protocol on the best performing sample (P-0.2), the weight losses after the sonication step and after the washing overnight in ethanol were evaluated and compared to the weight changes observed following the same procedure but using chloroform as a solvent, the latter being the standard solvent used to evaluate the insoluble fraction of crosslinked polymers. The values obtained for ethanol show results very similar to those obtained for chloroform, suggesting the good efficiency of ethanol as washing solvent. (Figure 5a). Furthermore, the UV-Vis spectrum of ethanol after the o/n washing step was collected showing no presence of released initiator. It can be assumed that the eventually unreacted compound, which could be potentially toxic, is completely extracted during the first sonication step or consumed during the post curing, with no further release after prolonged washing, confirming the efficacy of the chosen post-printing protocol.

### 3.3. Cell Viability of the 3D Printed Wells

Once selected, the sample with the best results (P-0.2) and the best washing protocol (sonication in ethanol), based on the results of the cytotoxic assay, we checked whether different sterilization methods could have some effects on biocompatibility, mechanical and optical characteristics of 3D printed wells. In particular, we tested three different sterilization methods: autoclave, ethanol, and UV-light. Half of the samples were incubated o/n in deionized water at room temperature [30], then they were sterilized, as previously described. The cells were seeded on both the P-0.2 24-like-wells and the PS conventional wells used as a control. After 72 h, live and dead assay was performed to check cell viability. 

**Figure 6:**
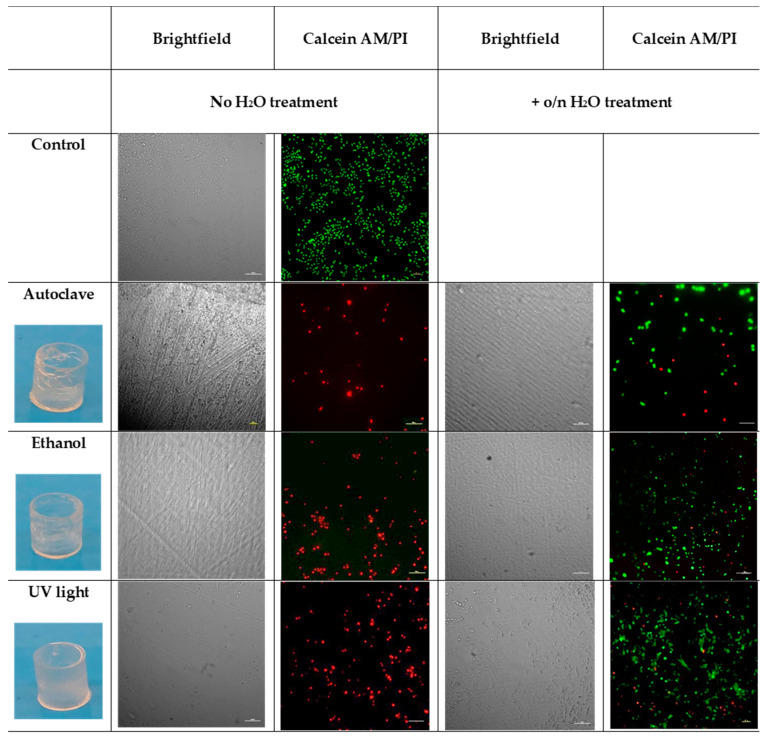
Images of the real 3D printed wells after the different sterilization methods. The survival status of the A549 cells on the P-0.2 3D printed wells, sterilized using autoclave, ethanol, and UV light with or without water pretreatment was evaluated by live/dead staining and phase-contrast image. The scale bar denotes 100 μm for all images.

As observed in Figure 6, the autoclaved wells lost their shape, therefore autoclave cannot be considered a suitable solution. Both the ethanol and the UV light sterilization methods do not damage the 3D printed wells; furthermore, live and dead assay showed that cells grow in both the samples. On the other hand, immersion in ethanol results in small defects in the transparency of the 3D printed wells if compared to the UV treated, making it difficult to visualize cells at the phase-contrast microscope. So, it resulted that the best sterilization method considering our goals was UV-sterilization.

## 4. Conclusions

In the present study, acrylate-based formulations were prepared for the fabrication of customized samples through a DLP 3D printer. In particular, 3D printed 96- and 24-wells and disk were made from commercial monomers, BEDA, HDDA, and PEGDA and using BAPO photoinitiator at both 0.2 wt.% and 1 wt.% of concentration. The 3D printed wells were tested by following typical assays as is common for PS wells, and the pros and the cons were analyzed for every material used for the cell culture experiments. In specific, by employing the suitable materials and implementing a series of post-processing steps, we demonstrated that the samples made of PEGDA with 0.2 wt.% of BAPO were the most appropriate for our goal. While somehow these results could be foreseen, here we evidenced the importance of the post-processing protocol. The most adequate one resulted in a washing protocol (sonication in ethanol) followed by sterilization under UV light. The cytotoxicity and the viability of A459 grown on 3D printed wells were investigated, showing no harm on cells, confirming that the photoinitiator (BAPO) and the acrylate unpolymerized can be successfully washed out. After two days of cultivation, the cells showed significant proliferation, proving their non-cytotoxicity. Compared to PS positive control, the PEGDA samples achieved viability around 50% after two days, results that can be further enhanced, for example by adding cell anchoring points. Our results evidence that components suitable for biomedical field can be fabricated with a conventional 3D printer, in lab condition and employing commercially available resins, not specifically designed for bio-applications, i.e., cheaper. Resultingly, what is more important is to establish a suitable post-processing method.

## Figures and Tables

**Table 1:** Composition and nomenclature of the home-made acrylate resins, average viscosity values at 25 °C, 3D printing settings used for each formulation with a slicing thickness of 50 µm, insoluble gel fraction (%) and acrylate conversion of the 3D printed objects.

Composition	Nomenclature	Viscosity	Exposure Time	Initial Exposure Time	Acrylate Conversion	Insoluble Fraction
		(Pa·s) ^a^	(s/layer)	(s/layer) ^b^	(%)	(%) ^c^
PEGDA + 0.2 wt.% BAPO	P-0.2	0.018	1.5	3	88	98
HDDA + 0.2 wt.% BAPO	H-0.2	0.019	1.5	3	85	99
BEDA + 0.2 wt.% BAPO	B-0.2	0.071	2	3	72	95
PEGDA + 1 wt.% BAPO	P-1	0.018	1	2	89	99
HDDA + 1 wt.% BAPO	H-1	0.020	1	3	89	99
BEDA + 1 wt.% BAPO	B-1	0.073	1.5	2	78	96

^a^ Average viscosity calculated between 1 and 100 s^−1^ of shear rate. ^b^ Irradiation times for the first three layers to guarantee an adequate adhesion of the parts. ^c^ Insoluble gel fraction of 3D printed parts calculated after 24 h of extraction in chloroform.
